# Gels for Water Remediation: Current Research and Perspectives

**DOI:** 10.3390/gels10090585

**Published:** 2024-09-12

**Authors:** Gabriela Buema, Adina-Elena Segneanu, Dumitru-Daniel Herea, Ioan Grozescu

**Affiliations:** 1 National Institute of Research and Development for Technical Physics, 47 Mangeron Boulevard, 700050 Iasi, Romania; gbuema@phys-iasi.ro; 2 Institute for Advanced Environmental Research, West University of Timişoara (ICAM–WUT), 4 Oituz Street, 300086 Timişoara, Romania; ioangrozescu@gmail.com

**Keywords:** sustainable economy, water remediation, wastewater treatment, hydrogels, aerogel materials, adsorption properties

## Abstract

The development of cost-effective and high-performance technologies for wastewater treatment is essential for achieving a sustainable economy. Among the various methods available for water remediation, adsorption is widely recognized as an effective and straightforward approach for removing a range of pollutants. Gel materials, particularly hydrogels and aerogels, have attracted significant research interest due to their unique properties. Hydrogels, for instance, are noted for their ability to be regenerated and reused, ease of separation and handling, and suitability for large-scale applications. Additionally, their low cost, high water absorption capacity, and contribution to environmental protection are important advantages. Aerogels, on the other hand, are distinguished by their low thermal conductivity, transparency, flexibility, high porosity, mechanical strength, light weight, large surface area, and ultralow dielectric constant. This review provides a comprehensive analysis of the current literature, highlighting gaps in knowledge regarding the classification, preparation, characterization, and key properties of these materials. The potential application of hydrogels and aerogels in water remediation, particularly in removing contaminants such as dyes, heavy metals, and various organic and inorganic pollutants, is also discussed.

## 1. Introduction

Water is one of the planet’s most vital resources and is essential for sustaining life. It is crucial for the survival of humans and animals, the healthy growth of plants, and the effective functioning of technological processes in both industry and agriculture, all of which rely on water and its quality.

In du Plessis’s publication [1], it is emphasized that the ongoing population growth, expansion of human activities, and intensified climate variations have significantly deteriorated water quality, posing threats to human health, ecosystem functioning, and socioeconomic development. The year 1990 is identified as the onset of global water degradation, which has placed immense pressure on an already diminishing resource. Since then, this degradation has accelerated, adversely affecting environmental quality as well as the health and safety of the global population [1]. Currently, more than two million tons of sewage, industrial, and agricultural waste are discharged into water bodies daily, with approximately 80% of it untreated, leading to numerous water-borne infectious diseases. The global challenges posed by water quality and the urgent need to secure safe drinking water, along with water for industrial and agricultural use, have necessitated immediate action and coordinated information efforts to prevent a severe water crisis with potentially disastrous global consequences. Consequently, the development of efficient water treatment and decontamination technologies has become an urgent priority, opening up a vast area of scientific research [1].

A World Bank report from 20 August 2019 [2], indicates that declining water quality is contributing to economic decline by up to a third in some countries, both developed and developing, particularly those heavily polluted, which threatens the well-being of people and the environment. World Bank Group President David Malpass emphasized that “clean water is a key factor for economic growth,” noting that deteriorating water quality is hindering economic progress, worsening health outcomes, reducing food production, and deepening poverty in many countries. He urged governments to take urgent action to combat water pollution and enable faster, more equitable, and environmentally sustainable growth [2].

In many countries, particularly those that are underdeveloped or still developing, the inefficiency of outdated technologies, lack of water pollution prevention measures, and high costs of advanced water treatment facilities render water remediation financially inaccessible. As a result, the problem of water contamination remains unresolved [3].

Water pollution has significant economic repercussions, negatively impacting tourism, property values, commercial fishing, recreational businesses, and other sectors reliant on clean water. Decontamination costs are prohibitively high, with only a few countries able to afford them. For example, nitrate removal systems in Minnesota have caused water supply costs to increase from 5 to 10 cents per 1000 gallons to over $4 per 1000 gallons. Cleaning polluted water bodies can cost billions of dollars upfront, although long-term treatment costs tend to decrease. However, the tourism industry alone loses nearly $1 billion annually due to reduced recreational fishing and boating activities. Additionally, properties near polluted waters with unpleasant appearances and odors suffer diminished value, particularly if no treatment measures are implemented [4].

A report from the National Intelligence Council [5] highlights the growing threat of water insecurity due to increasing demand and decreasing supply driven by ineffective treatment measures, corruption, and mismanagement. These issues jeopardize global economic growth, political stability, and interstate relations, especially in regions where water resources are shared, potentially leading to conflicts as water security diminishes and geopolitical competition intensifies. As the demand for clean water is expected to rise by 20% to 50% by 2050, driven by economic growth, rising incomes, and the increasing need for water-intensive goods and services, the water crisis could exacerbate existing social divisions and trigger conflicts between societal groups and industrial sectors, heightening political instability. As of 2020, approximately 2.1 billion people worldwide lack access to safe drinking water or reliable water services, a figure that could increase alarmingly in the coming years [5].

There are numerous techniques and technologies available for water remediation, whether for making water potable, purifying it, or removing pollutants. Each method has its own set of advantages and disadvantages. As a result, significant global efforts are being made to discover new materials and techniques for water treatment. Traditional methods—such as physical, biological, chemical, and membrane-based techniques—not only tend to be expensive with low efficiency, but they also often generate additional waste, like sludge, which incurs further costs for its disposal.

Recent research has identified more advanced water treatment methods, particularly those involving the adsorption of pollutants onto the surfaces of single, functionalized, or advanced composite materials. The emerging concept of the circular economy has also led to the discovery and use of waste materials with excellent adsorbent properties, which have been successfully employed in wastewater treatment. A key advantage of these new techniques is that they do not produce sludge.

Based on their unique properties and recent research findings, gels—used in various forms and combinations—have shown great promise in wastewater treatment processes. Adsorption has proven to be the most effective method for treating wastewater and is applicable to both composite materials derived from waste products, cost-effective and readily available materials, magnetic nanoparticles, and different types of gels. 

### 1.1. Hydrogel Materials

Hydrogel materials are well known for their ability to be regenerated and reused, ease of separation and handling, and suitability for large-scale applications [6]. These materials are also characterized by their low cost, high water absorption capacity, and environmental protection benefits [7].

In a review by Zhang and colleagues [7], several synthesis techniques for hydrogel materials are discussed. Based on crosslinking methods, hydrogels are categorized into three types: chemically crosslinked, physically crosslinked, and double-network hydrogels, with detailed descriptions provided for each method. The authors note that physical crosslinking is a simpler and faster process compared to chemical crosslinking. However, hydrogels produced through physical crosslinking tend to have poor mechanical properties. To enhance the adsorption performance of hydrogels, it is crucial to improve their mechanical properties, recyclability, and stability for future practical applications [7].

Ahmed [8], in a review paper, presents a comprehensive classification of hydrogels, their preparation methods, physical and chemical characterization techniques, and potential applications. Generally, hydrogels are defined as networks of crosslinked polymer chains that are highly saturated with water yet do not dissolve in it [8]. They can be synthesized through a single-step process, such as the simultaneous polymerization and crosslinking of multifunctional monomers, or through more complex procedures. Hydrogels can be made from natural polymers like collagen, gelatin, starch, alginate, and agarose, or from the chemical polymerization of synthetic monomers or their combinations. The basic process for hydrogel preparation involves polymerization through the reaction of hydrophilic monomers with multifunctional crosslinking agents in the presence of an initiator [8].

Hydrogels are classified based on the following criteria:

(i) polymer composition, which includes homopolymer hydrogels with a reticulated skeletal structure derived from a single monomer species and copolymer hydrogels based on two or more monomer species.
(ii) physical structure and chemical composition, which can lead to amorphous, semi-crystalline, or crystalline hydrogels.
(iii) types of crosslinking, which are categorized according to the physical or chemical nature of the crosslinking junctions.
(iv) physical appearance: hydrogels can be found in the form of matrices, films, or microspheres, depending on the polymerization method.
(v) electric charge of the network, leading to neutral, ionic, ampholytic (containing both acidic and basic groups), and zwitterionic hydrogels (containing both anionic and cationic groups in each structural unit).

A notable property of hydrogels is their ability to either expand and retain large volumes of water or shrink in the presence of water, responding reversibly to environmental conditions and physical stimuli such as temperature, light, pressure, electric or magnetic fields, chemical factors, pH, solvent composition, and molecular species. Hydrogels have a wide range of applications, including in agriculture, drug delivery, coal extraction, food additives, regenerative medicine, and biosensors. One of the recent applications of hydrogels, leveraging their high porosity, is the adsorption of pollutants from wastewater, particularly dyes and heavy metals [8].

Numerous research groups have conducted comprehensive reviews on the performance of various hydrogel materials for water remediation. For instance, Jiang et al. [9] examined carbon dot–hydrogel composite materials, focusing on the synthesis methods of carbon dots within hydrogels and their applications in detecting and removing water pollutants. Similarly, Le and colleagues [10] summarized the effectiveness of cellulose-based hydrogels for water treatment and purification. Additionally, Ahmaruzzaman et al. [11] reviewed polymeric hydrogels for wastewater depollution, while Kumari and co-workers [12] analyzed gum acacia-based hydrogels and their composites for wastewater treatment. 

In their review, Radoor and collaborators [13] emphasized the effectiveness of alginate- and cellulose-based hydrogels, highlighting their affordability, eco-friendliness, and efficiency in water remediation. These hydrogels are biodegradable and biocompatible and demonstrate strong capabilities in removing dyes, heavy metals, oils, pharmaceutical contaminants, and pesticides from wastewater. However, the review also addressed the limitations of alginate and cellulose-based hydrogels, such as low mechanical strength and poor stability. To overcome these drawbacks, the authors proposed strategies to enhance the physicochemical properties of these hydrogels by functionalizing them with nanomaterials and magnetic particles or reinforcing them with fibers.

One example involves enhancing the mechanical resistance by incorporating nanocrystals and chitosan fibers [13]. Additionally, hydrogels can be classified based on their crosslinking process, which significantly influences their elasticity, swelling, porosity, and mechanical strength. Factors such as the type of crosslinking agent, duration and degree of crosslinking, and density and state of crosslinking all play a crucial role in determining these properties [13]. For instance, a high degree of crosslinking can decrease the adsorption efficiency of a hydrogel by reducing the flexibility of the polymer chains. Ideally, a hydrogel with strong adsorbent properties should exhibit good flexibility, tunable porosity and surface charge, thermomechanical stability, high pH stability, and a large specific surface area. Smart hydrogels, designed to respond to external stimuli such as temperature, light, pH, electric and magnetic fields, and ionic strength, have been developed for water treatment applications [13]. These hydrogels must maintain good functionality and a stable 3D structure in various solvents. An effective adsorbent should also have a large specific surface area, high selectivity and removal efficiency, good regeneration capacity, low production and operating costs, and the ability to function over a wide pH range. The limitations of conventional hydrogels, such as their limited selectivity and weak regeneration capacity, can be addressed by using nanotechnology to create nanostructured hydrogels. These nanoadsorbents provide a large specific surface area and active functional groups and are particularly effective at removing both organic and inorganic contaminants from water. In addition, natural adsorbents like clay, zeolite, and coal hold significant promise from both economic and ecological perspectives [13].

### 1.2. Aerogels Materials

Aerogels are advanced solid materials derived from organic or inorganic precursors, known for their exceptional properties, such as low thermal conductivity, transparency, flexibility, high porosity and mechanical strength, low weight and density, large surface area, and ultralow dielectric constant [14]. However, their production is costly [14] and requires complex synthesis methods.

The swelling properties of aerogels are crucial for their adsorption performance. Enhanced swelling enables the rapid achievement of adsorption equilibrium by expanding the open structure of the aerogel, thereby increasing the availability of active sites for adsorption [15].

In their review, Garg and collaborators [16] highlight aerogels as advanced materials with large specific surface areas, making them ideal for various applications due to their excellent thermal, mechanical, and chemical properties. Aerogels can be derived from natural, synthetic, or hybrid materials and can be functionalized with adsorbent nanoparticles. This functionalization significantly enhances their specific surface area, thereby increasing their adsorption capacity—particularly through chemisorption—and expanding the range of pollutants they can remove from wastewater.

The pore size of aerogels can be extremely small, less than 2 nm, or within the mesoporous range of 2 nm to 50 nm. Their density, a key factor influencing their functionality, is remarkably low, often less than 4.2 kg/m^3^, which is much lighter than air (1.225 kg/m^3^) and water (1000 kg/m^3^) [16]. Aerogels are typically blue but can change color when exposed to infrared or ultraviolet light, enabling them to act as radiation sensors [16]. Their mechanical strength is closely tied to their density, and they exhibit excellent thermal resistance and elasticity, often returning to their original shape after the stress is removed, particularly in graphene-based aerogels.

Silica-based aerogels, with their specific particle size, can delay sound wave transmission, a property that is influenced by the frequency of the sound. Their proven biocompatibility makes aerogels valuable for biomedical applications [16]. The preparation of aerogels involves several steps, including the preparation of the colloidal gel matrix, addition of optional materials, and gelation in the presence of a crosslinking agent. The resulting material is then hardened and dried to remove the liquid component, producing an aerogel [16]. The aerogel synthesis process is influenced by various physical and chemical factors, such as precursor concentration (which impacts surface area and porosity), solvent type, catalyst amount and type, pH, temperature, gelation time, and surfactant selection—all of which affect the final properties of the aerogel. 

Aerogels can be made from natural organic and inorganic materials, as well as synthetic polymers like polyethylene glycol, polyimide, and phenolic resin [16]. Hybrid aerogels, which combine natural materials with synthetic polymers, are often created to enhance mechanical strength and expand their applications. However, the preparation of hybrid aerogels is a lengthy, time- and energy-intensive process, which is a significant drawback [16].

The main preparation methods and classifications of the gels are summarized in Table 1.

Ganesamoorthy and collaborators [17] outlined various preparation methods and types of aerogels. Aerogels can generally be classified as organic, inorganic, or hybrid materials. Depending on the drying method used during preparation, they can be categorized as aerogels (supercritical dried), xerogels (dried at ambient pressure), or cryogels (freeze-dried). Additionally, aerogels can exhibit different surface properties, such as being hydrophilic, hydrophobic, amphiphilic, oleophilic, or oleophobic, depending on their polarity and surface functionality [17].

The effectiveness of various aerogel materials in water depollution has been extensively reviewed. Liu and collaborators [19] discussed montmorillonite-based aerogels for environmental remediation. Shao and co-workers examined the application of carbon aerogel-based materials in persulfate activation for water treatment [20]. More recently, Nguyen and collaborators [21] reviewed bacterial cellulose-based aerogels, focusing on synthesis methods, regeneration, cost analysis, challenges, and future prospects for water treatment. Gao and colleagues [22] presented the use of graphene-based aerogels in water and air treatment. Additionally, Baimenov and co-workers [23] explored the synthesis methods and properties of Ti_3_C_2_T_x_ MXene/3D hydrogel and aerogel composites, highlighting their potential applications in water remediation [23].

An important point to note is that hydrogels and aerogels are interchangeable forms of matter. The gel state itself is neither fully liquid nor fully solid in its physical properties, which can result in unique relaxation behaviors that are not observed in purely solid or liquid states. Due to this characteristic, hydrogels can exhibit noticeable volume changes when subjected to external stimuli, such as temperature, solvent quality, pH, or electric fields [24].

### 1.3. Characterization of Gel Materials

Gulrez and collaborators [25] outlined several techniques for characterizing hydrogels, focusing on their solubility and swelling properties in water, which are critical for their applications. The authors described various physical and chemical analysis methods, including Fourier Transform Infrared Spectroscopy (FTIR) for identifying the chemical structure of the hydrogel in comparison to its precursors; Scanning Electron Microscopy (SEM) for examining the surface topography and network structure of the hydrogel; gel permeation chromatography with Multi-Angle Laser Light Scattering (GPC-MALLS) for determining the molecular distribution and polymer system parameters; sol-gel analysis to estimate parameters such as crosslinking yield, degradation, and gelation dose, and their correlation with certain physicochemical properties [25].

Additionally, techniques such as differential scanning calorimetry (DSC), nuclear magnetic resonance (NMR), thermogravimetric analysis (TGA), and X-ray diffraction (XRD) have been used to quantify the amount of free and bound water in hydrogels [25]. Rheological investigations also play a crucial role in the characterization of hydrogels [26,27].

Garg and colleagues [16] highlighted that the physical and chemical characterization of aerogels involves several methods, including thermogravimetric analysis (TGA) to assess thermal stability; optical property analysis across visible, near-infrared, and infrared wavelengths to study spectral reflectance, transmission, and emissions; density, porosity, and hardness measurements; flammability testing to determine the temperature range in which the aerogel remains functional; structural characterization and particle size estimation using Scanning Electron Microscopy (SEM) and Transmission Electron Microscopy (TEM); and Small-Angle X-ray Scattering (SAXS) for estimating average pore size [16].

Furthermore, X-ray diffraction (XRD), Brunauer-Emmett-Teller (BET) surface area analysis, and X-ray photoelectron spectroscopy (XPS) have also been employed to characterize both hydrogels and aerogels [26,28,29,30,31].

The primary aim of this work is to review recent research on the potential applications of various gel types, specifically hydrogels, in wastewater treatment. This review also highlights the effectiveness of certain aerogel materials for water remediation. It demonstrates the significant capabilities of both hydrogels and aerogels in removing a range of contaminants from water. These include heavy metals (such as chromium, copper, cadmium, nickel, and lead), dyes (including rhodamine B, methyl orange, methylene blue, congo red, crystal violet, and malachite green), as well as emerging pollutants like antibiotics, anti-inflammatory drugs, pesticides, and other substances related to water disinfection.

## 2. Applications of Gels in Water Remediation

### 2.1. Applications of Hydrogels in Water Remediation 

#### 2.1.1. Removal of Organic Dyes

Organic dyes, which are commonly used in industries such as textiles, food, and paints, are known for their high color persistence, resistance to microbial degradation, and photolytic stability. These characteristics make their removal from wastewater particularly challenging. To address some of the limitations associated with current removal methods, Marullo and colleagues [32] synthesized a range of supramolecular ionic liquid gels based on diimidazolium salts with naturally occurring or biomass-derived anions. They investigated the effectiveness of these gels in treating water contaminated with rhodamine B and methyl orange dyes [32]. Their comparative analysis revealed the following:

- [p-C_12_][Mal]/[PF_6_] material removed 71% methyl orange dye after 48 h and 92% rhodamine B after 24 h [32].;
- for rhodamine B dye, removal efficiencies higher than 90% were achieved by [p-C_12_][Fum]/[PF_6_] (95% in 6 h), [p-C_12_][Mal]/[NTf_2_] (93% in 15 h), and [p-C_12_][Fum]/[NTf_2_] (97% in 6 h) materials [32];
- [p-C_12_][Mal]/[SCN] and [p-C_12_][Fum]/[SCN] can remove rhodamine B dye of 31% (72 h) and 36% (48 h), respectively [32].

Roa and collaborators [33] conducted a study where they prepared a series of hydrogel materials, considering three parameters: free-radical initiator (APS), crosslinking agent (MBA), and the amount of cellulose nanofibers (CNF). The samples, labeled Hy01-Hy12, were evaluated for their ability to treat water contaminated with the dye methyl orange. They investigated the effects of contact time, pH, CNF concentration, and initial dye concentration on adsorption. The results indicated that the Hy01 material exhibited a higher adsorption capacity, reaching 1379 mg/g at a concentration of 2000 mg/L. Regeneration and reusability tests using HCl as the eluent demonstrated that the material could be reused effectively for up to three cycles.

Yang and co-workers [34] tested porous hydrogel beads for their ability to adsorb rhodamine B dye from aqueous solutions. These beads achieved a maximum adsorption capacity of 1648.3 mg/g, and the study confirmed the successful reusability of the hydrogel.

**Figure 1:**
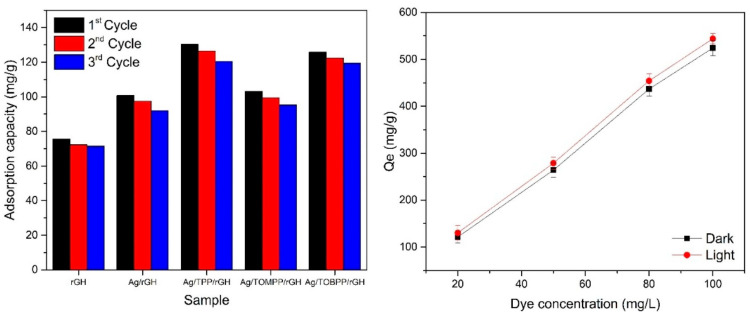
Reusability studies of the hydrogels on the adsorption of methylene blue (**left**); effect of the initial methylene blue concentration on the adsorption of Ag/TPP/rGH (**right**). Source [35] with permission from the Elsevier and Copyright Clearance Center.

Zheng and colleagues [35] prepared a reduced graphene oxide hydrogel modified with silver nanoparticles and various porphyrin complexes. Their results showed that Ag/TPP/rGH had an enhanced adsorption capacity for methylene blue dye, with a maximum of 130.37 mg/g, compared to other materials investigated. The hydrogel was found to be reusable for up to three cycles (Figure 1 left). Further studies on the Ag/TPP/rGH hydrogel assessed the influence of the initial dye concentration under both dark and light conditions (Figure 1 right) [35].

Hou and colleagues [36] synthesized various types of hydrogels using cyclodextrin polymer (P-CD) and ferrocene-modified polyacrylic acid (PAA-Fc). The resulting materials, designated as Gel-A, Gel-B, and Gel-C, were evaluated for their effectiveness in removing bisphenol A and methylene blue. The results indicated the following:

- Gel-A material could remove 23.61 mg/g of Bisphenol A and 19.74 mg/g of methylene blue [36];
- Gel-B removed 20.55 mg/g and 21.6 mg/g of bisphenol A and methylene blue, respectively [36];
- Gel-C presented a slightly decreased adsorption capacity for bisphenol A (18.39 mg/g) compared with Gel-B; however, its adsorption capacity for methylene blue was improved compared to Gel-A hydrogels (24.64 mg/g) [36].

In their study, Moharrami and Motamedi [37] developed a novel hydrogel based on a nanocomposite derived from agricultural waste. They used cellulose nanocrystals (CNCs) functionalized with magnetite nanoparticles (Fe_3_O_4_) and employed sugar beet pulp, an agricultural by-product, which was processed through acid hydrolysis with enzyme assistance. CNCs functionalized with Fe_3_O_4_ nanoparticles were incorporated into an acrylic acid hydrogel (starch-g-(AMPS-co-AA)) to create a composite (MCNC). This new hydrogel nanocomposite demonstrated high efficiency and environmental friendliness as a nano-adsorbent for selectively removing cationic dyes, such as methylene blue and crystal violet, showing excellent absorption capacity and reusability [37].

In the context of water remediation, Peng and colleagues [38] developed a hydrogel composed of cellulose and clay that exhibited high efficiency in removing dyes, particularly methylene blue, with removal rates between 96.6% and 98%. The adsorption process was found to be physical and followed pseudo-second-order kinetics and the Langmuir isotherm model [38].

**Figure 2:**
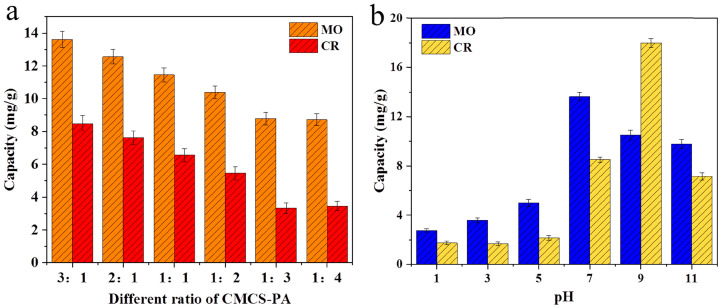
(**a**) Adsorption capacity of the CMCS-PA composite hydrogels for methyl orange and congo red dyes in different ratios; (**b**) Adsorption capacity for methyl orange and congo red dyes of the CMCS-PA (3:1 ratio) at different solution pH values. Source [39], with permission from Elsevier and the Copyright Clearance Center.

Han and co-workers [39] synthesized carboxymethyl chitosan/phytic acid (CMCS-PA) composite hydrogels in various ratios and tested their effectiveness for removing methyl orange and congo red dyes (Figure 2a). The hydrogel with a 3:1 ratio showed superior adsorption capacity compared to the other formulations. Further investigations on the effect of solution pH revealed that the maximum adsorption capacity for methyl orange was 13.62 mg/g at pH 7, and for congo red was 17.97 mg/g at pH 9 (Figure 2b). The reusability study indicated that the CMCS-PA (3:1) hydrogel could be effectively reused for five cycles, maintaining 88.1% efficiency for methyl orange and 89.7% for congo red [39].

Recently, a nanocomposite hydrogel consisting of bentonite nanoclay (BNC) impregnated in chitosan-gelatin (CS-g-GEL/BNC) has been employed for the removal of congo red dye [40]. The newly developed hydrogel achieved a maximum adsorption capacity of 453.876 mg/g. Furthermore, reusability tests demonstrated that the hydrogel maintained effective removal performance for congo red dye over five cycles [40].

**Figure 3:**
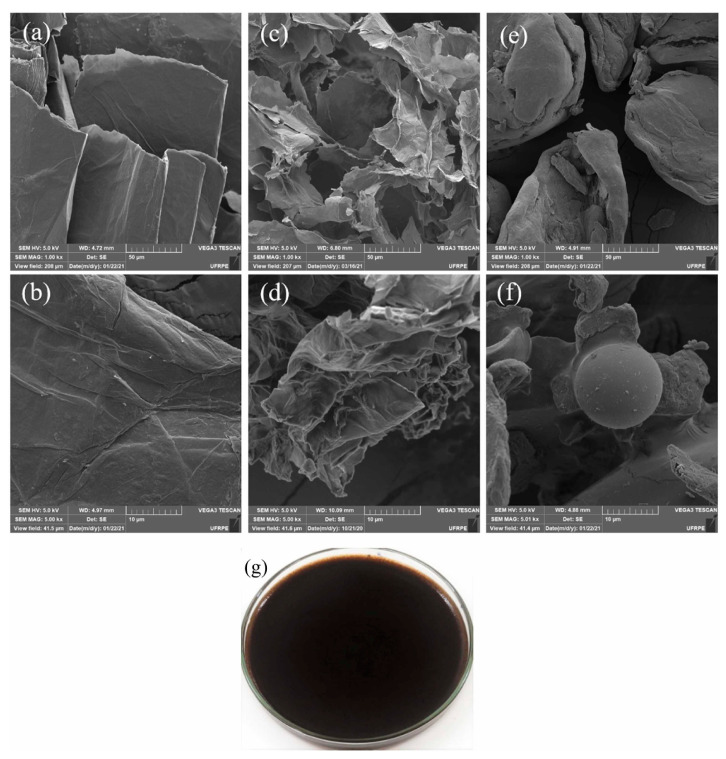
(**a**,**b**) SEM images of GO; (**c**,**d**) SEM images of freeze-dried agar-GO; (**e**,**f**) SEM images of powdered pure agar; (**g**) Photograph of the produced hydrogel. Source [41], with permission from Elsevier and the Copyright Clearance Center.

In another study, de Araujo and colleagues [41] synthesized a compound made from graphene oxide and agar biopolymer, referred to as agar-GO, for treating textile wastewater. The performance of this material was tested with dyes, including Nile blue A, methylene blue, malachite green, and basic fuchsin. Representative SEM images of graphene oxide (GO), agar, and the synthesized agar-GO are shown in Figure 3 [41]. These results confirmed that agar-GO effectively removed the targeted dyes [41].

Lv and colleagues [42] successfully utilized a composite hydrogel to remove contaminants such as methylene blue, rhodamine B, methyl orange, and indigo red dyes. The hydrogel achieved maximum adsorption capacities of 349.68 mg/g for methylene blue, 325.42 mg/g for methyl orange, 306.08 mg/g for indigo red, and 332.85 mg/g for rhodamine B, all measured at pH 7 and 25 °C. The proposed adsorption mechanism involves electrostatic and hydrogen bonding interactions [42]. Additionally, the authors investigated the catalytic activity of composite hydrogels loaded with Ni-NPs for the degradation of 4-nitrophenol, yielding promising results [42].

**Figure 4:**
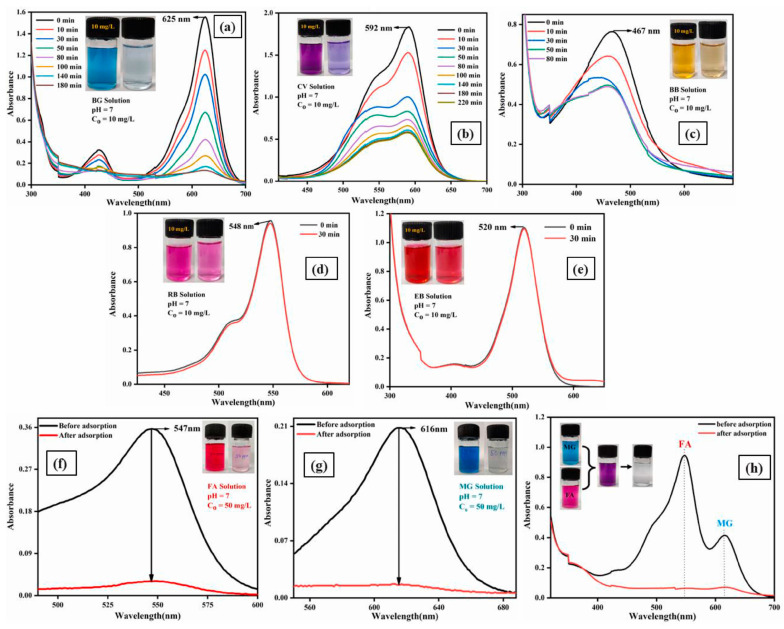
Changes in the absorption spectrum: (**a**) brilliant green (BG); (**b**) crystal violet (CV); (**c**) bismark brown (BB); (**d**) rose bengal (RB); (**e**) eosin blue (EB); (**f**) fuchsin acid (FA); (**g**) malachite green (MG); (**h**) the mixture of malachite green (MG) and fuchsin acid (FA). Source [43] with permission from the Elsevier and Copyright Clearance Center.

Another study focused on the removal of malachite green (MG) and fuchsin acid (FA) dyes using a pullulan/CMC hydrogel [43]. The hydrogel’s adsorption performance was evaluated by varying several parameters: initial dye concentrations from 10 to 50 mg/L, adsorbent dosages of 0.1 to 0.35 g, temperatures ranging from 30 °C to 70 °C, and pH values of 2, 5, 7, 9, and 11. The influence of ionic strength was also tested [43]. Furthermore, the PA/CMC hydrogel was assessed for its effectiveness in removing various dyes, including brilliant green (BG), crystal violet (CV), Bismark brown (BB), rose bengal (RB), and eosin blue (Figure 4a–e) [43]. The hydrogel performance in a binary system of MG and FA was evaluated (Figure 4h) [43]. The results indicate that the PA/CMC hydrogel is particularly effective for removing malachite green and fuchsin acid dyes.

The effectiveness of the nanocomposite hydrogel (TG-cl-PAA/Fe_3_O_4_) for dye removal was assessed for three dyes: malachite green, thioflavin T, and rhodamine B [44]. The study was conducted in single, binary, and ternary systems. In the ternary system, the hydrogel demonstrated a maximum adsorption capacity of 642.9 mg/g for malachite green, 413.6 mg/g for thioflavin T, and 552.6 mg/g for rhodamine B [44].

Altaleb [45] synthesized a sulfonated copolymer hydrogel and investigated its capacity for adsorbing crystal violet, achieving an adsorption capacity of 518.49 mg/g. 

In a separate study, a hydrogel based on katira gum crosslinked with poly(acrylic acid-co-N-vinyl imidazole) was evaluated for its effectiveness in treating water contaminated with four different dyes [46]. The hydrogel demonstrated adsorption capacities of 331.5 mg/g for methylene blue, 286.01 mg/g for methyl violet, 201.53 mg/g for tartrazine, and 273.5 mg/g for carmoisine-A [46].

Lamkhao and colleagues [47] recently synthesized a porous zinc oxide/polyacrylamide hydrogel composite photocatalyst (ZnO/PAM) using a photoassisted polymerization method. Their research focused on two main areas: (a) adsorption studies of methylene blue and methyl orange dyes, examining the effects of the initial solution pH (ranging from 2 to 10), contact time (10–720 min), adsorption kinetics, and isotherms; and (b) assessing the photocatalytic efficiency of the material using real wastewater samples. Wastewater was sourced from the Mae Kha Canal in Chiang Mai Province, Thailand, and from an industrial cleaning chemical manufacturer. The ZnO/PAM material demonstrated good stability and reusability, achieving degradation efficiencies of up to 81.55% for methylene blue and 75.4% for methyl orange after 50 cycles.

Das and Patel [48] developed a lemon peel-chitosan hydrogel composite as a potential adsorbent for various dyes. The hydrogel effectively removed 24.984 mg/g of safranin O, 24.788 mg/g of methylene blue, 24.862 mg/g of basic fuchsin, 23.483 mg/g of toluidine blue, 24.409 mg/g of brilliant green, and 24.726 mg/g of crystal violet. After six regeneration cycles using 10% acetic acid, the hydrogel retained over 80% of its removal efficiency for all tested dyes.

Huaman and collaborators [49] synthesized magnetic hydrogels made of Fe_3_O_4_/poly(HEMA-co-AMPS) for the removal of methylene blue. The in situ synthesis of Fe_3_O_4_ particles within the hydrogels yielded a maximum adsorption capacity of 445.35 mg/g, as determined by the Langmuir model. The composite hydrogel also showed strong reusability, maintaining 94% of its performance after 10 consecutive cycles.

#### 2.1.2. Heavy Metals and Other Contaminants Removal

Azzeddine and co-workers [50] aimed to utilize shrimp waste from El Jadida City, Morocco, to produce chitosan hydrogel beads crosslinked with epichlorohydrin for fluoride removal. The study found that non-crosslinked chitosan gel beads removed 12.64 mg/g of fluoride, while crosslinked chitosan gel beads increased the removal capacity to 16.59 mg/g.

Jaques and collaborators [51] developed hydrogels containing glycerol-based macromonomers for treating water contaminated with mercury and caffeine. The three hydrogels synthesized exhibited adsorption capacities of 46.26 mg/g, 63.04 mg/g, and 91.55 mg/g for caffeine, and 312.25 mg/g, 369.75 mg/g, and 385.74 mg/g for mercury ions. Additionally, they conducted experiments to detect caffeine and mercury in surface water from Paranoá Lake, achieving recovery rates between 96.37% and 99.66% for caffeine and over 99% for mercury across all tested materials.

Ming Li and colleagues [52] investigated cellulose-based hybrid hydrogel beads incorporating carbon dots for the adsorption and detection of mercury ions in water. The hydrogel demonstrated a maximum adsorption capacity of 290.70 mg/g and retained over 80% of its initial capacity after five regeneration cycles. The beads also maintained sensitive and selective sensing abilities for mercury, even after two months of storage.

Chu and co-workers [53] developed a hydrogel with significant adsorption capacities for mercury and chromium, measuring 846.7 mg/g and 289.5 mg/g, respectively. The adsorption process was attributed to electrostatic attraction and covalent bond coordination.

Thiyagarajan’s research group [54] focused on CaCO_3_ nanocomposite hydrogels for the adsorption and removal of chromium (VI) ions from aqueous solutions. Their study examined the effects of pH (2–8), adsorbent dosage (0.1–0.5 g), initial chromium concentration (50–500 ppm), and contact time (60–360 min). The findings revealed the following:

- increasing pH from 2 to 3 improved the removal efficiency, but further increases led to a decline.
- raising the adsorbent dosage from 0.1 g to 0.4 g enhanced removal efficiency, with no significant changes at 0.5 g.
- equilibrium was reached after 240 min of contact time.
- higher initial chromium (VI) concentrations reduced the removal efficiency to 75%.
- HCl was identified as the most effective desorption agent compared to HNO_3_ or EDTA.

Ouass and collaborators [55] synthesized sodium polyacrylate hydrogel powder to remove chromium (III) ions from aqueous solutions. They examined factors like pH, adsorbent dosage, initial chromium concentration, contact time, and temperature, finding a maximum adsorption capacity of 269 mg/g.

Zhao and Li [56] created a floatable carboxymethyl cellulose/polyacrylamide hydrogel (PAM/CMC/DDM) for zinc ion removal. Additionally, they designed zinc-loaded hydrogels (PAM/CMC/DDM-ZnO composites) that functioned as photocatalysts.

Li and co-workers [57] explored palladium removal using acrolein crosslinked chitosan hydrogels, achieving high adsorption capacities (~478.47 mg/g at 303 K, ~502.5 mg/g at 313 K, and 505.05 mg/g at 323 K).

Chen and collaborators [58] utilized wastepaper mixed with acrylamide double-network hydrogel (WP/PAM) to treat water contaminated with copper and methylene blue dye. The WP/PAM material exhibited a maximum adsorption capacity of 142.2 mg/g for copper and 1714.5 mg/g for methylene blue at 25 °C. At 35 °C, these capacities increased to 145.3 mg/g for copper and 1734.9 mg/g for methylene blue. The adsorbent could be reused for up to six cycles.

Kamal and colleagues [59] developed a hydrogel through the graft copolymerization of methacrylic acid, acrylamide, and 2-acrylamido-2-methyl-1-propanesulfonic acid onto sodium alginate. The hydrogel effectively removed 87% of cadmium and 97.5% of methylene blue dye using only 0.15 g/100 mL, with maximum adsorption capacities of 526 mg/g for cadmium and 131.5 mg/g for methylene blue.

**Figure 5:**
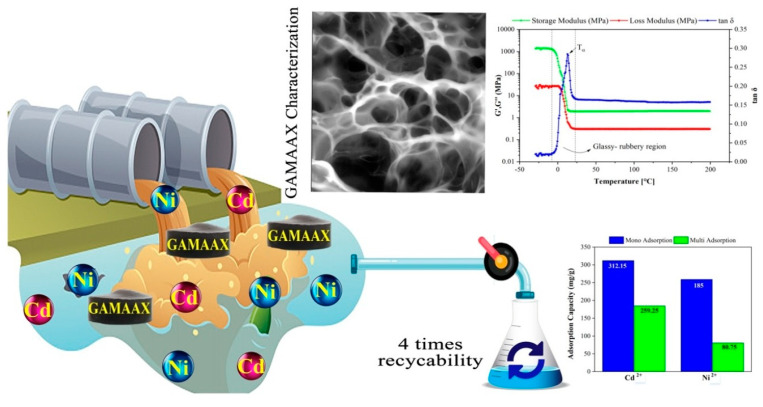
Schematic diagram of the study. Source [60], with permission from Elsevier and the Copyright Clearance Center.

Jafarigol and colleagues [60] developed a hydrogel material composed of poly(acrylamide-co-acrylic acid)/xanthan gum (XG) incorporated with graphene oxide (GAMAAX) for the removal of cadmium and nickel ions. The material was tested in both single and binary systems. In the single system, the adsorption capacities were 312.15 mg/g for cadmium and 185 mg/g for nickel. In the binary system, the material achieved removal capacities of 259.25 mg/g for cadmium and 80.75 mg/g for nickel. Additionally, the hydrogel demonstrated the ability to undergo multiple regeneration cycles. The schematic diagram of the study is depicted in Figure 5.

Chen and colleagues [61] developed a hydrogel based on poly(gamma-glutamic acid) (PGA) for the removal of copper, chromium, and zinc ions. The study initially focused on the hydrogel’s capacity to remove copper ions, examining adsorption kinetics (emphasizing pseudo-first and pseudo-second-order models), adsorption isotherms (using Langmuir and Freundlich models), adsorption thermodynamics, pH influence, adsorbent dosage, and reusability over five cycles. The hydrogel exhibited a maximum adsorption capacity of 21.41 mg/g for copper ions, with higher capacities of 212.3 mg/g for zinc and 198.63 mg/g for chromium. 

In another study, Mu and co-workers [62] introduced a hydrogel adsorbent specifically for lead removal, conducting experiments across varying contact times (15 to 720 min) and temperatures (15 °C to 35 °C). The hydrogel demonstrated an adsorption capacity of 170.31 mg/g and maintained good performance after four regeneration cycles. 

Zhang and colleagues [63] synthesized a hydrogel (T-PMADA) through the copolymerization of acrylic acid (AA) and methacrylamide dopamine (MADA) with Ti_3_C_2_ MXene, achieving maximum adsorption capacities of 609.9 mg/g for lead ions and 250.2 mg/g for cadmium ions. Additionally, this hydrogel proved effective for the photocatalytic degradation of methyl orange, methylene blue, and eosin Y dyes.

**Figure 6:**
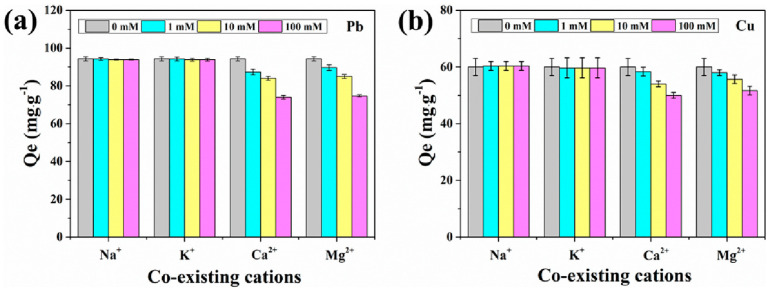
Effect of co-existing cations on the adsorption of (**a**) Pb(II) and (**b**) Cu(II). Source [64], with permission from Elsevier and the Copyright Clearance Center.

Sun and co-workers [64] developed a chitosan/cellulose phosphonate composite hydrogel (CS/MCCP), which showed maximum adsorption capacities of 211.42 mg/g for lead and 74.29 mg/g for copper. The study also investigated the effects of co-existing cations on the adsorption process (Figure 6).

Niu and collaborators [65] developed a hydrogel (PAM/PAA/PDMTM) composed of polyacrylamide, polyacrylic acid, and poly(2,4-dimercapto-6-mercaptopropenyl-1,3,5-triazine), and tested its efficacy in removing copper, cadmium, and lead ions. The hydrogel demonstrated adsorption capacities of 92.33 mg/g for copper, 110.08 mg/g for cadmium, and 200.97 mg/g for lead ions. Additionally, the study showed that PAM/PAA/PDMTM could also effectively remove lead from the soil.

Ma and co-workers [66] explored the removal of lead, cadmium, and copper ions using a hydrogel material synthesized from electrolytic manganese slag combined with polyacrylic acid-polyacrylamide. The findings indicated that this hydrogel is a suitable candidate for water remediation, with adsorption capacities of 153.85 mg/g for lead, 113.63 mg/g for cadmium, and 54.35 mg/g for copper ions.

Yang and co-workers [67] introduced a hydrogel that shows promise for treating water contaminated with lead, methylene blue dye, and crystal violet dye. The maximum adsorption capacities, calculated using the Langmuir isotherm model, were 1021.85 mg/g for lead ions, 890.90 mg/g for methylene blue, and 827.54 mg/g for crystal violet. These results were obtained under conditions of an initial pollutant concentration of 100 mg/L for lead, 25 mg/L for methylene blue and crystal violet, pH 6 ± 0.2, adsorbent dosage of 0.2 g/L, 298 K, with a contact time of 12 h for lead and 48 h for the dyes.

**Figure 7:**
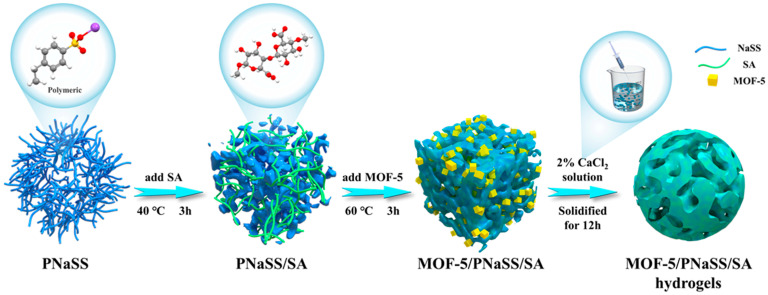
Schematic diagram of the synthesis of hydrogel. Source [68], with permission from Elsevier and the Copyright Clearance Center.

In another study, Zhang and co-workers [68] fabricated a novel hydrogel, MOF-5/PNaSS/SA, and thoroughly investigated its ability to remove lead and tetracycline. The schematic diagram of the synthesis of hydrogel is presented in Figure 7. Various operational parameters were considered to study the adsorption behavior, and the Langmuir isotherm model provided the best fit for the data. The maximum adsorption capacities based on this model are summarized in Table 2.

Tetracycline and norfloxacin, two antibiotics, were effectively removed using a hydrogel that integrates ZIF-67 into chitosan and reduced graphene oxide (rGO), resulting in the rGO@ZIF-67@CS material [69]. The adsorption capabilities of various materials, including CS, rGO, ZIF-67, ZIF-67@CS, and rGO@ZIF-67@CS, were compared using 0.015 g of each material in a 30 mL solution with an initial concentration of 50 mg/L at 303 K. The rGO@ZIF-67@CS hydrogel exhibited the highest adsorption capacities, achieving 93.46 mg/g for tetracycline and 94.71 mg/g for norfloxacin. In comparison, ZIF-67@CS showed adsorption capacities of 88.29 mg/g for tetracycline and 89.17 mg/g for norfloxacin, while ZIF-67 alone achieved 87.77 mg/g and 87.87 mg/g, respectively. rGO displayed lower adsorption capacities of 56.99 mg/g for tetracycline and 44.64 mg/g for norfloxacin, and the lowest capacities were observed with CS, at 34.79 mg/g for tetracycline and 25.42 mg/g for norfloxacin. According to the Langmuir model, the rGO@ZIF-67@CS hydrogel exhibited high maximum adsorption capacities of 1685.26 mg/g for tetracycline (at 313 K) and 1890.3 mg/g for norfloxacin (at 293 K) [69].

Another study investigated a nanocomposite hydrogel composed of magnetic composite nanoparticles synthesized by encapsulating magnetite in a polystyrene-co-polymethacrylic acid mini-emulsion, which demonstrated the ability to adsorb cesium, cobalt, and strontium ions [70]. The adsorption capacity increased significantly as the initial pollutant concentration was increased from 50 mg/L to 600 mg/L, achieving increases from 8.49 mg/g to 53.37 mg/g for cesium, 11.17 mg/g to 80.69 mg/g for cobalt, and 10.75 mg/g to 65.35 mg/g for strontium [70].

In a recent study by Dai and co-workers [71], a phytic acid-functionalized polyamidoxime/alginate hydrogel (PAG) was synthesized through a one-step hydrothermal reaction and applied for uranium removal from water [71]. The hydrogel exhibited a maximum adsorption capacity of 218.34 mg/g, as determined by the Langmuir isotherm model. The study also included an investigation of the hydrogel’s adsorption selectivity and reusability, with HNO3 proving to be the most effective desorption eluent [71].

Other hydrogel adsorbents including Mt/PE-@SA, Nt/PE-@SA, and Bd/PE-@SA have also been explored for uranium removal [72]. These hydrogels were synthesized using sodium alginate (SA), polyethylene (PE), and natural clay minerals such as montmorillonite (Mt), nontronite (Nt), and beidellite (Bd). Among these, Nt/PE-@SA exhibited the highest adsorption capacity (133.3 mg/g), followed by Bd/PE-@SA (87.7 mg/g) and Mt/PE-@SA (50.5 mg/g). The study also examined the adsorption selectivity of these hydrogels for uranium in the presence of co-existing metal ions, including copper, chromium (III), manganese, nickel, cadmium, and lead, revealing a strong affinity for uranium in all three hydrogels [72].

Salahuddin and collaborators [73] developed two hybrid nanocomposites (CNF/alginate and MNP–CNF/alginate hydrogel beads) and evaluated their effectiveness in adsorbing aluminum, potassium, selenium, sodium, sulfur, and vanadium. The materials were designed using an alginate polymer matrix reinforced with magnetic nanoparticles decorated with cellulose nanofibers; the results of these experiments are summarized in Table 3 [73].

A β-cyclodextrin-MOF-based porous hydrogel was evaluated for its effectiveness in removing Au^3+^, Ag^+^, and Pb^2+^ ions [74]. The hydrogel exhibited adsorption capacities of 316.4 mg/g for Au^3+^ and 60.9 mg/g for Ag^+^. Notably, it demonstrated a high removal capacity for Pb^2+^, achieving 414.2 mg/g [74].

In another study, Guo and colleagues [75] developed a CeCuO_x_ nanocatalytic gel and assessed its water disinfection capabilities [75]. The material showed high disinfection efficiency without producing harmful byproducts, even at low dosages [75]. The gel was tested on bacteria such as *E. coli*, *B. subtilis*, and *P. aeruginosa*, showing consistent performance across 20 consecutive disinfection cycles, maintaining 90% to 100% efficiency depending on the cycle count [75]. Their research highlighted the gel’s potential for effective water treatment [75].

Table 4 presents the selection of gel materials used for the removal of various dyes from aqueous media.

The results illustrate that different dye pollutants exhibit different activities for the maximum adsorption capacity. Numerous studies have reported that the adsorption performance of these pollutants depends on the contact time, temperature, and solution pH. An increase in adsorption performance with increasing pH values was observed, which was attributed to changes in the chemical properties of the pollutant and gel surface. The maximum adsorption occurred within the pH range of 6–7.8 [32,58,67]. 

Contact time is another decisive factor influencing pollutant adsorption. In the initial phase, the removal rate increases rapidly due to the availability of numerous vacant sites. Adsorption equilibrium is reached at high values of time as a result of the fact that the adsorbent still has numerous vacant sites [32,67]. 

Temperature is a parameter that affects molecular interactions and solubility. An increase in temperature enhances the adsorption capacity, likely due to a higher number of available adsorption sites on the adsorbent surface or an increased diffusion rate of the contaminant [58].

For gel materials, a high initial concentration of pollutants increases the removal efficiency due to the higher likelihood of collisions between adsorption sites and contaminant molecules [32,58,67].

Last but not least, the type of adsorbate, the physicochemical characteristics, in particular surface area, pore size, pore distribution, and pore surface chemistry have a significant effect on the performance of the adsorption process.

### 2.2. Applications of Aerogels in Water Remediation 

#### 2.2.1. Organic Dye Removal 

Zhang and colleagues [76] developed reduced-graphene-oxide/rare-earth-metal-oxide aerogels (RGO/REMO) for the removal of rhodamine B. They synthesized three types of aerogels: RGO/Pr_2_O_3_, RGO/Ce_2_O_3_, and RGO/Nd_2_O_3_, achieving adsorption capacities of 226 mg/g, 235.7 mg/g, and 243.4 mg/g, respectively [76].

**Figure 8:**
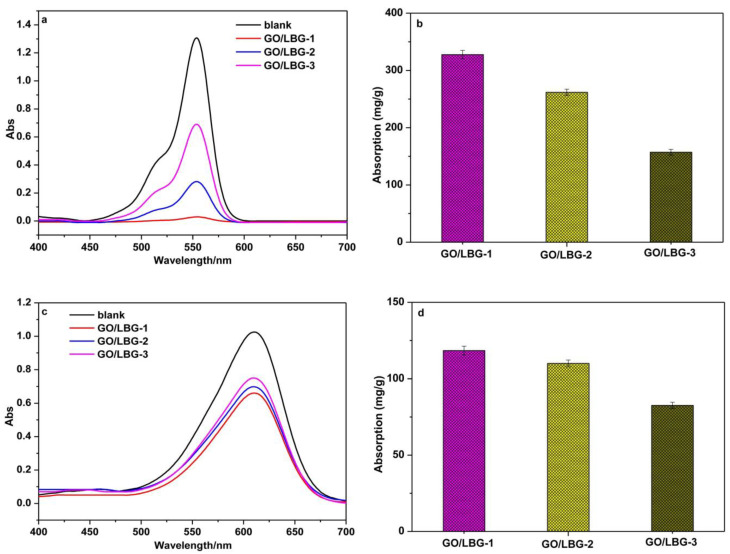
(**a**) UV–vis absorption spectra of Rhodamine B, solution adsorbed by graphene oxide/locust bean gum (GO/LBG) aerogels with GO/LBG mass ratios of 1:4 (GO/LBG-1), 1:8 (GO/LBG-2), 1:16 (GO/LBG-3) for 24 h, (**b**) the maximum adsorption quantity on Rhodamine B of GO/LBG-1, GO/LBG-2, and GO/LBG-3 aerogels, (**c**) UV–vis absorption spectra of Indigo carmine solution adsorbed by GO/LBG-1, GO/LBG-2, and GO/LBG-3 aerogels for 24 h, and (**d**) the maximum adsorption quantity on Indigo carmine of GO/LBG-1, GO/LBG-2, and GO/LBG-3 aerogels. Source [88] with permission from the Elsevier and Copyright Clearance Center.

In a separate study, Li and co-workers [88] created graphene oxide/locust bean gum hybrid aerogels (GO/LBG) with varying mass ratios of GO to LBG—1/4 (GO/LBG-1), 1/8 (GO/LBG-2), and 1/16 (GO/LBG-3)—for the removal of rhodamine B and indigo carmine [88]. The results indicated that the aerogel with a 1/4 mass ratio was the most effective at removing both dyes (Figure 8). Notably, the aerogel exhibited a higher maximum adsorption capacity for rhodamine B compared to indigo carmine (Table 5) [88].

In another study, He and collaborators [77] explored the use of pectin/graphene oxide aerogels (PTGA) for the removal of rhodamine B and methyl orange. The aerogel containing 15 mg/mL of pectin demonstrated superior adsorption capacity compared to other aerogel variants [77].

Yadav and co-workers [78] developed a methionine-functionalized graphene oxide/chitosan polymer nanocomposite aerogel (meth-GO/CH) for the removal of dyes and heavy metals. The targeted pollutants included rhodamine B, crystal violet, nickel ions, and copper ions. The maximum adsorption capacities calculated from the Langmuir isotherm model were 46.511 mg/g for rhodamine B, 243.902 mg/g for crystal violet, 66.225 mg/g for nickel, and 149.253 mg/g for copper [78].

Tao and colleagues [80] prepared graphene oxide-montmorillonite/sodium alginate (GO-MMT/SA) aerogel beads for methylene blue removal, achieving a maximum adsorption capacity of 150.66 mg/g. The adsorption mechanism likely involves electrostatic and π-π interactions [80].

**Figure 9:**
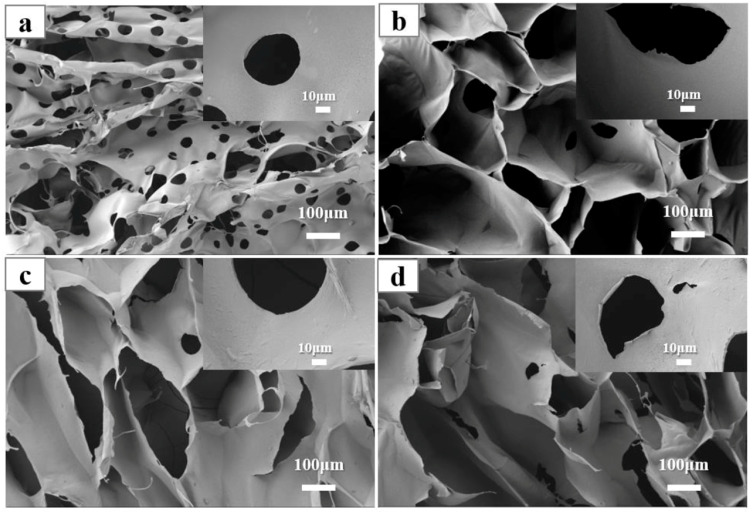
SEM images of CMC/O (**a**), CMC/CNF–C (**b**), CMC/CNFs (**c**), and CMC/CNWs (**d**). Source [81], with permission from Elsevier and the Copyright Clearance Center.

Zhang and collaborators [81] synthesized four types of cellulose-based aerogels—CMC/O, CMC/CNF–C, CMC/CNFs, and CMC/CNWs—using polyethylene glycol diglycidyl ether (PEGDE) as a crosslinking agent. The representative SEM images are depicted in Figure 9. The material was tested for methylene blue removal, and after comparing its chemical/physical structure, mechanical properties, adsorbate dispersion, and adsorption capacity, CMC/CNF–C was selected for further dye removal studies. According to the Langmuir isotherm model, this aerogel exhibited a maximum adsorption capacity of 917.43 mg/g [81].

Jing and co-workers [82] developed a carboxymethyl cellulose (CMC)-based aerogel (CMC/PSA) incorporating p-styrene sulfonate (SSS) and acrylic acid (AA) as organic monomers for methylene blue removal. This aerogel demonstrated a high adsorption capacity of 925.9 mg/g at 323 K [82].

Wang and colleagues [83] synthesized a composite aerogel from graphene oxide and nanocellulose (GO/nanocellulose) for the removal of methylene blue and tetracycline. The Langmuir isotherm model indicated maximum adsorption capacities of 112.2 mg/g for methylene blue and 47.3 mg/g for tetracycline. The aerogel also showed a high reusability rate, with 98% efficiency for methylene blue and 97% efficiency for tetracycline after three cycles using ethanol [83].

Tang and co-workers [84] investigated a composite aerogel, prepared via a one-step reaction and freeze-drying, for the removal of heavy metal ions (copper, lead, cadmium, iron) and methylene blue dye. The aerogel demonstrated effective water treatment capabilities with adsorption capacities of 314.6 mg/g for methylene blue, 446 mg/g for copper, 222 mg/g for lead, 267 mg/g for cadmium, and 600 mg/g for iron [84].

An MXene/carbon foam hybrid aerogel (MCF) was created by synthesizing Ti_3_C_2_T_X_-MXene with melamine foam (MF) and was used for removing methylene blue and congo red dyes. The aerogel achieved adsorption capacities of 356.97 mg/g for methylene blue and 647.75 mg/g for congo red [85].

Hu and collaborators [86] prepared a composite aerogel and tested it for methylene blue and congo red dye removal. According to the Langmuir isotherm model, the aerogel had maximum adsorption capacities of 182.6 mg/g for methylene blue and 590.6 mg/g for congo red [86].

**Figure 10:**
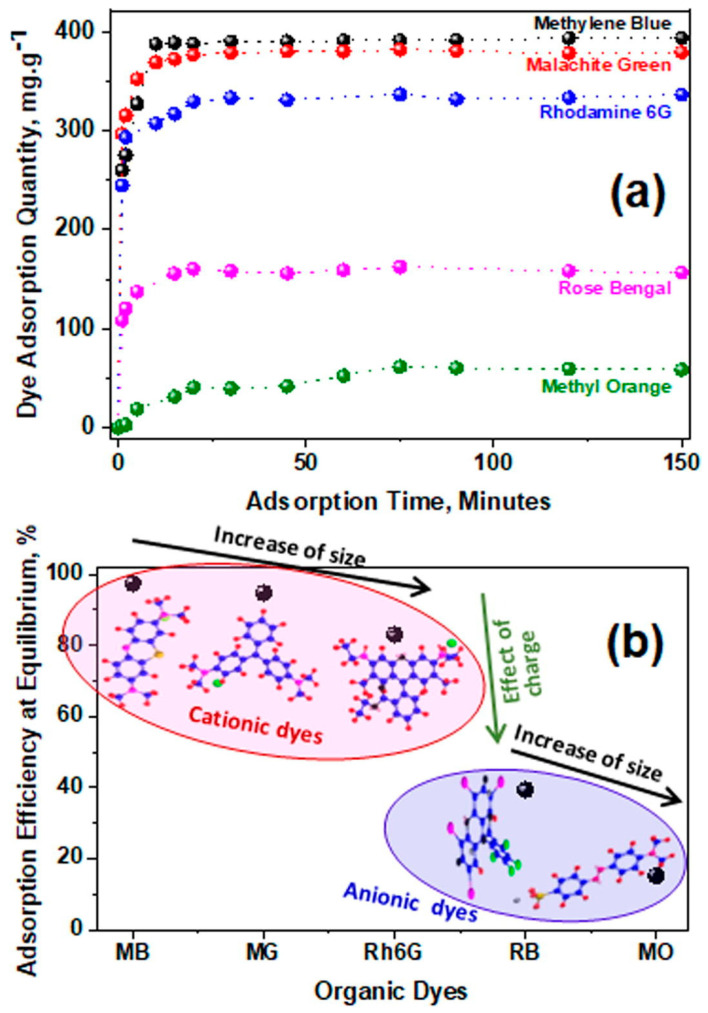
(**a**) Adsorption quantity of variable organic dyes, methylene blue (MB), malachite green (MG), rhodamine 6G (Rh6G), Rose bengal (RB), and methyl orange (MO) by CGA as a function of time. (**b**) Dye adsorption efficiency of CGA at equilibrium, along with the demonstration of the molecular structure of each dye. Source [89] with permission from the Elsevier and Copyright Clearance Center.

Joshi and co-workers [89] developed a cellulose-graphene oxide composite aerogel (CGA) using cellulose extracted from Pomelo peels and graphene oxide (GO). This aerogel was evaluated for the removal of various dyes, including methylene blue, malachite green, rhodamine 6G, rose bengal, and methyl orange. The results showed that the aerogel had a higher adsorption capacity for cationic dyes compared to anionic dyes and that the adsorption efficiency decreased with increasing molecular size of the dye molecules [89]. The dye adsorption quantity vs. adsorption time is presented in Figure 10a and the adsorption efficiency at equilibrium vs. organic dyes is presented in Figure 10b.

Wang and collaborators [79] developed an egg yolk/zeolitic imidazolate framework-8/crosslinked polyacrylic acid aerogel (EY/ZIF-8/CLPAA) for the removal of various dyes, including malachite green, crystal violet, rhodamine B, and methyl orange. The aerogel exhibited impressive adsorption capacities, with values of 2338 mg/g for malachite green, 489 mg/g for crystal violet, 447 mg/g for methyl orange, and 299 mg/g for rhodamine B [79].

In a study by Gong and colleagues [87], a composite aerogel made from sugarcane bagasse–bentonite and sodium alginate was utilized for crystal violet removal. The aerogel demonstrated a high adsorption capacity of 839.9 mg/g [87].

#### 2.2.2. Removal of Various Pollutants (Organic Dyes, Antibiotics, Heavy Metals)

Wang and co-workers [90] prepared a composite aerogel, ZIF-8@ANF/PVA, by growing zeolitic imidazolate frameworks-8 (ZIF-8) in situ on aramid nanofibers (ANF) and poly(vinyl alcohol) (PVA) networks. This aerogel was tested for various pollutants, including heavy metals (Cr(VI)), dyes (congo red), and an antibiotic (tetracycline). The material showed notable adsorption capacities: 230.5 mg/g for Cr(VI), 319.5 mg/g for tetracycline, and 547.0 mg/g for congo red [90].

**Figure 11:**
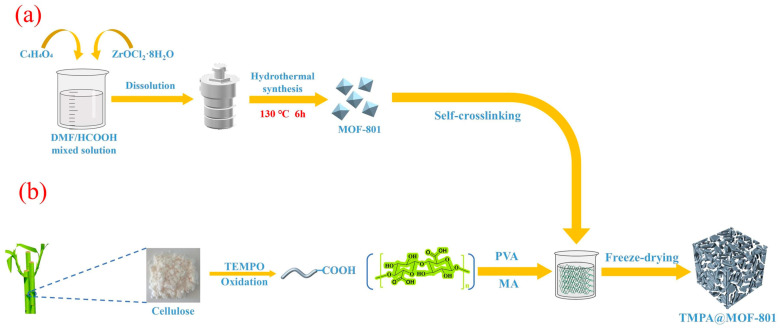
Schematic diagram of the synthesis of MOF-801 (**a**) and TMPA@MOF-801 aerogel (**b**). Source [91], with permission from Elsevier and the Copyright Clearance Center.

Zhao and collaborators [91] introduced a novel composite aerogel by combining the metal-organic framework MOF-801 with nanocellulose for the first time. The schematic diagram of the synthesis of aerogel is presented in Figure 11. The aerogel was evaluated for Cr(VI) removal, achieving an adsorption capacity of 350.64 mg/g according to the Langmuir isotherm model [91].

Li and colleagues [92] developed a graphene oxide nanosheets/chitosan composite aerogel and evaluated its effectiveness for removing the anionic dyes methyl orange and bromophenol blue. The aerogel exhibited maximum adsorption capacities of 179.21 mg/g for methyl orange and 236.42 mg/g for bromophenol blue [92].

Ma and co-workers [93] prepared a carbon aerogel from waste paper and used it for tetracycline removal. The aerogel demonstrated maximum adsorption capacities of 384.6 mg/g at 25 °C, 413.2 mg/g at 35 °C, and 432.9 mg/g at 45 °C. Regeneration studies revealed that the aerogel could be reused for 10 cycles with only a 10.4% reduction in the adsorption capacity [93].

Hadi Yatimzade and colleagues [94] investigated the removal of ibuprofen using a magnetic carbon aerogel (MCA). They employed the Central Composite Design (CCD) method to assess the effects of parameters such as solution pH, contact time, and adsorbent dosage. According to the Langmuir isotherm model, the maximum adsorption capacity of the MCA was 46.94 mg/g. Additionally, the MCA was shown to be reusable for up to five cycles [94].

Tartrazine dye removal was studied using a hybrid sodium alginate/graphene oxide aerogel (SA-GO) by [95]. The SA-GO aerogel achieved a maximum adsorption capacity of 420.36 mg/g, outperforming the sodium alginate (SA) aerogel. The study also explored the aerogel’s ability to remove ibuprofen [95].

Yin and collaborators [96] synthesized a guanidine-functionalized sericin/nanocellulose aerogel (GSNA) for water disinfection and copper ion removal. Their results indicated that the GSNA was effective for these applications. Additionally, the GSNA was utilized as a membrane filter for the simultaneous removal of E. coli bacteria and copper ions [96].

Nguyen and colleagues [97] developed a magnetic Fe_3_O_4_/graphene aerogel composite using co-precipitation and partial reduction methods. This composite exhibited a maximum adsorption capacity of approximately 42.918 mg/g for the pesticide 2,4-dichlorophenoxyacetic acid [97].

## 3. Adsorption Mechanism of Gels for Water Remediation

Zhu and colleagues [98] provided a concise review focusing on the adsorption mechanisms of hydrogel based materials for the removal of heavy metals and organic dyes. They explored various potential interactions, including electrostatic forces, hydrogen bonding, π-π interactions, ion exchange, surface complexation, and coordination/chelation, offering a detailed explanation of each mechanism.

Similarly, Ahmaruzzaman and co-workers [11] reviewed the adsorption mechanisms of pollutants by hydrogels, detailing interactions such as hydrophobic forces, ion exchange, electrostatic interactions, π-π interactions, and hydrogen bonding.

The adsorption mechanism for aerogel-type materials is primarily chemisorption with ion exchange as a mediating process, following the Langmuir isotherm described mainly by a pseudo-second kinetic order [16].

For a comprehensive overview of the adsorption mechanisms for different metal ions (chromium, copper, cadmium, nickel, lead, mercury, and zinc) using various aerogels, refer to the tabular presentation in the review by Ihsanullah and collaborators [15].

## 4. Critical Analysis of Gel Materials

### 4.1. Aerogel-Type Materials 

Aerogels display remarkable characteristics (3-D porosity, high surface area, unique thermal, mechanical, and chemical properties, lightweight, low cost, ecological, flexible, and recyclable materials) and exhibit notable performance, especially in the adsorption processes of various pollutants from wastewater [16,99].

Adsorption efficacy and reusability are key factors that influence the application potential of aerogel materials in industrial water treatment processes. Thus, the adsorbent performance depends on several factors, including the pH, contact time, specific surface, and ionic medium. In addition, the recycle/reuse process of aerogel-type materials is time-consuming compared that with of conventional adsorbents [16,99].

Despite their outstanding sorption–desorption capacity and good regeneration, gaps affecting their implementation in industrial use are related to economic efficiency. Consequently, comprehensive techno-economic analyses are required to improve the experimental conditions for aerogel preparation, mainly during the drying step (freeze or supercritical drying) [16,99].

### 4.2. Hydrogel-Type Materials

Hydrogels are materials composed of a unique matrix of hydrophilic natural or synthetic polymers with physical or chemical crosslinks. Due to their outstanding features (high water absorption capacity and structural versatility at various physical or chemical modifications), hydrogels are used in different fields [18].

Recent research has addressed the development of tailored synthetic hydrogels with high structural flexibility, thermal and mechanical stability, and enhanced adsorption capacity for various organic and inorganic pollutants compared to natural hydrogels [62].

Special attention has been paid to replacing synthetic polymers, which are linked to several environmental issues, such as slow degradation, pollution, and greenhouse gas emissions. Consequently, biopolymers, which are derived from renewable resources and are often biodegradable, are being considered as potential alternatives for mitigating the environmental impact of synthetic polymers. However, a thorough and ongoing assessment of their overall sustainability, including their environmental footprint throughout their entire lifecycle—production, use, and disposal—is essential [18,62,100].

## 5. Conclusions

This review examines the effectiveness of various gels, including hydrogels and aerogel types, for water remediation. It provides a brief overview of their classification, preparation methods, characterization techniques, and key properties.

Both hydrogels and aerogels have demonstrated significant potential for removing a wide array of organic and inorganic contaminants from water. Advancements in this area could enable the scale-up of laboratory innovations for broader wastewater treatment applications. This review highlights the considerable potential of these adsorbents in addressing diverse contaminants, including heavy metals (such as chromium, copper, cadmium, nickel, and lead), dyes (like rhodamine B, methyl orange, methylene blue, Congo red, crystal violet, and malachite green), as well as emerging pollutants (including antibiotics, anti-inflammatory drugs, and pesticides), and for water disinfection.

Future research should focus on improving the adsorption capabilities of these gels and exploring underutilized waste materials to develop new, highly efficient gels. Continued progress in sustainable and innovative water treatment techniques is essential for ecosystem preservation.

Future studies will also address the application of aerogels in oil/water separation, highlighting another significant and intriguing use of aerogel materials beyond their current applications in heavy metal, dye, and antibiotic removal.

## Figures and Tables

**Table 1:** Aerogels and hydrogels: classification and synthesis routes.

Name	Preparation Methods	Preparation Steps	Classification		Ref.
			Based on Composition	Based on Drying Gel	
Aerogels	sol-gel	gel preparation	organic	aerogels (super critically dried)	[17]
		gel aging	inorganic	xerogels (ambient pressure-dried)	
		gel drying	hybrid	cryogels (freeze-dried)	
Hydrogels	polymerization and crosslinking of multifunctional monomers, copolymerization, free radical polymerization, genetic engineering, irradiation, photo-polymerization, reversible addition-fragmentation chain transfer (RAFT), atom transfer radical polymerization (ATRP), nitroxidemediated polymerization (NMP), etc		**first-generation hydrogels** (a) polymerization of water-soluble monomers (poly(hydroxyalkyl methacrylate)s), (b) hydrogels based on crosslinking of water-soluble synthetic polymers poly(vinyl alcohol (PVA) and poly(ethylene glycol) (PEG), (c) hydrogels based on cellulose		[18]
			**second-generation hydrogels** (a) temperature-sensitive hydrogels based on PEG-polyester block copolymers, poly(N-isopropylacrylamide (PNIPAM)), etc. (b) in situ forming hydrogels based on other stimuli.		
			**third-generation hydrogels** (a) stereocomplexed hydrogels poly(ethylene glycol)-poly(lactic acid) interaction (b) hydrogels crosslinked by other physical interactions (e.g., cyclodextrins). (c) stimuli-responsive hydrogels or “smart hydrogels”		

**Table 2:** Maximum adsorption capacity from the Langmuir model [68].

Hydrogel Sample	Pollutant	Adsorption Capacity, mg/g
MOF-5/PNaSS/SA	lead	243.6 (288 K)
		239.3 (298 K)
		225.2 (308 K)
	tetracycline	209.4 (288 K)
		201.3 (298 K)
		188.4 (308 K)

**Table 3:** Adsorption capacity of CNF/Alginate and MNP–CNF/Alginate [73].

Contaminant	Adsorption Capacity of CNF/Alginate vs. Adsorption Capacity of MNP–CNF/Alginate (mg/g)
aluminum	1.22/22
potassium	6.6/13.2
selenium	14.3/19
sodium	8.8/11.1
sulfur	9.8/13.7
vanadium	11.1/44.4

**Table 4:** Various gel materials tested for dye removal.

Dye	Gel Materials	Adsorption Capacity	Contact Time (h)	Temperature (K)	Ref.
rhodamine B	[p-C_12_][Fum]/[NTf_2_]	97%	6		[32]
	[p-C_12_][Fum]/[PF_6_]	95%	6		[32]
	[p-C_12_][Mal]/[NTf_2_]	93%	15		[32]
	[p-C_12_][Mal]/[PF_6_]	92%	24		[32]
	[p-C_12_][Fum]/[SCN]	36%	48		[32]
	[p-C_12_][Mal]/[SCN]	31%	72		[32]
	SA/AOPIM-1	1648.3 mg/g	-	-	[34]
	quaternary ammonium ionic liquids (DM)	332.85 mg/g		298.15	[42]
	TG-cl-PAA/Fe_3_O_4_	552.6 mg/g			[44]
	RGO/Pr_2_O_3_	226 mg/g			[76]
	RGO/Ce_2_O	235.7 mg/g			[76]
	RGO/Nd_2_O_3_	243.4 mg/g			[76]
	PTGA	719 mg/g			[77]
	meth-GO/CH	46.511 mg/g			[78]
	EY/ZIF-8/CLPAA	299 mg/g			[79]
methyl orange	[p-C_12_][Mal]/[PF_6_]	71%	48		[32]
	Hy01	1379.0 mg/g			[33]
	CMCS-PA	13.62 mg/g			[39]
	quaternary ammonium ionic liquids (DM)	325.42		298.15	[42]
	PTGA	419 mg/g			[77]
	EY/ZIF-8/CLPAA	447 mg/g			[79]
methylene blue	Ag/TPP/rGH	130.37 mg/g			[35]
	quaternary ammonium ionic liquids (DM)	349.68 mg/g		298.15	[42]
	Fe_3_O_4_/poly(HEMA-co-AMPS)	445.35 mg/g			[49]
	WP/PAM	1714.5 mg/g		298	[58]
	WP/PAM	1734.9 mg/g		308	[58]
	SAH	97.5%			[59]
	SLS/DTPA@ZIF-8	890.90 mg/g	48		[67]
	GO-MMT/SA	150.66 mg/g			[80]
	CMC/CNF–C	917.43 mg/g			[81]
	CMC/PSA	925.9 mg/g		323	[82]
	GO/nanocellulose	112.2 mg/g			[83]
	PDA/TOCNF	314.6 mg/g			[84]
	MCF	356.97 mg/g			[85]
	CGK	182.6 mg/g			[86]
crystal violet	SLS/DTPA@ZIF-8	827.54 mg/g	48		[67]
	meth-GO/CH	243.902 mg/g			[78]
	EY/ZIF-8/CLPAA	489 mg/g			[79]
	SCB–Ben/SA	839.9 mg/g			[87]
congo red	CMCS-PA	17.97 mg/g			[39]
	CS-g-GEL/BNC	453.876 mg/g			[40]
	MCF	647.75 mg/g			[85]
	CGK	590.6 mg/g			[86]
malachite green	TG-cl-PAA/Fe_3_O_4_	642.9 mg/g			[44]
	EY/ZIF-8/CLPAA	2338 mg/g			[79]

**Table 5:** Maximum adsorption capacity values [88].

Pollutant	Aerogel Sample	Adsorption Capacity, mg/g
Rhodamine B	GO/LBG-1	514.5
	GO/LBG-2	468.2
	GO/LBG-3	288.1
Indigo carmine	GO/LBG-1	134.6
	GO/LBG-2	129.8
	GO/LBG-3	95.3

## Data Availability

Data described in the manuscript will be made publicly and freely available without restriction.

## References

[1] du Plessis A. (2022). Persistent degradation: Global water quality challenges and required actions. One Earth.

[2] (2019). Worsening Water Quality Reducing Economic Growth by a Third in Some Countries: World Bank. https://www.worldbank.org/en/news/press-release/2019/08/20/worsening-water-quality-reducing-economic-growth-by-a-third-in-some-countries.

[3] Anantadjaya S.P.D., Rahmadani M.S., Satiri, Nawangwulan I.M., Rachmat T.A. The economic impact of water pollution. Proceedings of the 2nd International Conference on Economics, Business and Social Sciences.

[4] https://www.epa.gov/nutrientpollution/effects-economy.

[5] https://www.dni.gov/index.php/gt2040-home/gt2040-deeper-looks/future-of-water.

[6] Weerasundara L., Gabriele B., Figoli A., Ok Y.S., Bundschuh J. (2021). Hydrogels: Novel materials for contaminant removal in water—A review. Crit. Rev. Environ. Sci. Technol..

[7] Zhang Z., Fu H., Li Z., Huang J., Xu Z., Lai Y., Qian X., Zhang S. (2022). Hydrogel materials for sustainable water resources harvesting & treatment: Synthesis, mechanism and applications. Chem. Eng. J..

[8] Ahmed E.M. (2015). Hydrogel: Preparation, characterization, and applications: A review. J. Adv. Res..

[9] Jiang M., Wang Y., Li J., Gao X. (2024). Review of carbon dot–hydrogel composite material as a future water-environmental regulator. Int. J. Biol. Macromol..

[10] Le V.T., Joo S.W., Berkani M., Mashifana T., Kamyab H., Wang C.Q., Vasseghian Y. (2023). Sustainable cellulose-based hydrogels for water treatment and purification. Ind. Crops Prod..

[11] Ahmaruzzaman M., Roy P., Bonilla-Petriciolet A., Badawi M., Ganachari S.V., Shetti N.P., Aminabhavi T.M. (2023). Polymeric hydrogels-based materials for wastewater treatment. Chemosphere.

[12] Kumari P., Kumar M., Kumar R., Kaushal D., Chauhan V., Thakur S., Shandilya P., Sharma P.P. (2024). Gum acacia based hydrogels and their composite for waste water treatment: A review. Int. J. Biol. Macromol..

[13] Radoor S., Karayil J., Jayakumar A., Kandel D.R., Kim J.T., Siengchin S., Lee J.W. (2024). Recent advances in cellulose- and alginate-based hydrogels for water and wastewater treatment: A review. Carbohydr. Polym..

[14] Zubair N.A., Abouzari-Lotf E., Mahmoud Nasef M., Abdullah E.C. (2019). Aerogel-based materials for adsorbent applications in material domains. E3S Web Conf..

[15] Ihsanullah I., Sajid M., Khan S., Bilal M. (2022). Aerogel-based adsorbents as emerging materials for the removal of heavy metals from water: Progress, challenges, and prospects. Sep. Purif. Technol..

[16] Garg S., Singh S., Shehata N., Sharma H., Samuel J., Khan N.A., Ramamurthy P.C., Singh J., Mubashir M., Bokhari A. (2023). Aerogels in wastewater treatment: A review. J. Taiwan Inst. Chem. Eng..

[17] Ganesamoorthy R., Vadivel V.K., Kumar R., Kushwaha O.S., Mamane H. (2021). Aerogels for water treatment: A review. J. Clean. Prod..

[18] Nikolić L.B., Zdravković A.S., Nikolić V.D., Ilić-Stojanović S.S. (2018). Synthetic hydrogels and their Impact on health and environment. Cellulose-Based Superabsorbent Hydrogels.

[19] Liu C., Li Z., Li B., Zhang H., Han J. (2023). Montmorillonite-based aerogels assisted environmental remediation. Appl. Clay Sci..

[20] Shao B.B., Xu Y.T., Liu Z.F., Wu T., Pan Y., Zhang X.S., He M., Ge L., Lu Y., Liu Y. (2023). Application of carbon aerogel-based materials in persulfate activation for water treatment: A review. J. Clean. Prod..

[21] Nguyen N.T.T., Nguyen L.M., Nguyen T.T.T., Nguyen D.T.C., Tran T.V. (2024). Synthesis strategies, regeneration, cost analysis, challenges and future prospects of bacterial cellulose-based aerogels for water treatment: A review. Chemosphere.

[22] Gao B., Feng X.B., Zhang Y.F., Zhou Z.X., Wei J.F., Qiao R., Bi F.K., Liu N., Zhang X.D. (2024). Graphene-based aerogels in water and air treatment: A review. Chem. Eng. J..

[23] Baimenov A., Daulbayev C., Poulopoulos S.G., Mochalin V.N. (2024). MXene filled hydrogel and aerogel composites. Mater. Today.

[24] Neethu T.M., Dubey P.K., Kaswala A.R. (2018). Prospects and Applications of Hydrogel Technology in Agriculture. Int. J. Curr. Microbiol. Appl. Sci..

[25] Gulrez S.K.H., Saphwan Al-Assaf S., Phillips G.O. (2011). Hydrogels: Methods of Preparation, Characterisation and Applications. Progress in Molecular and Environmental Bioengineering—From Analysis and Modeling to Technology Applications.

[26] Chronopoulou L., Di Nitto A., Papi M., Parolini O., Falconi M., Teti G., Muttini A., Lattanzi W., Palmieri V., Ciasca G. (2021). Biosynthesis and physico-chemical characterization of high performing peptide hydrogels@graphene oxide composites. Colloids Surf. B Biointerfaces.

[27] Zhang H.L., Zhang B., Cai C.Y., Zhang K.M., Wang Y., Wang Y., Yang Y.M., Wu Y.G., Ba X.W., Hoogenboom R. (2024). Water-dispersible X-ray scintillators enabling coating and blending with polymer materials for multiple applications. Nat. Commun..

[28] Zhao W.T., Zhu J., Wei W., Ma L.R., Zhu J.J., Xie J.M. (2018). Comparative study of modified/non-modified aluminum and silica aerogels for anionic dye adsorption performance. RSC Adv..

[29] Yi B., Li T.J., Yang B.G., Chen S.R., Zhao J.Y., Zhao P.C., Zhang K.Y., Wang Y., Wang Z.K., Bian L.M. (2024). Surface hydrophobization of hydrogels via interface dynamics-induced network reconfiguration. Nat. Commun..

[30] Wen Y., Chen X., Yan H., Lin Q. (2022). Comparative Study of Physicochemical Properties of Alginate Composite Hydrogels Prepared by the Physical Blending and Electrostatic Assembly Methods. Gels.

[31] Shiri M., Hosseinzadeh M., Shiri S., Javanshir S. (2024). Adsorbent based on MOF-5/cellulose aerogel composite for adsorption of organic dyes from wastewater. Sci. Rep..

[32] Marullo S., Rizzo C., Dintcheva N.T., Giannici F., D’Anna F. (2018). Ionic liquids gels: Soft materials for environmental remediation. J. Colloid Interface Sci..

[33] Roa K., Tapiero Y., Thotiyl M.O., Sánchez J. (2021). Hydrogels Based on Poly([2-(acryloxy)ethyl] Trimethylammonium Chloride) and Nanocellulose Applied to Remove Methyl Orange Dye from Water. Polymers.

[34] Yang X., Zhang X., Feng X., Xu B., Du C., Zhang E., Shan M., Zhang Y. (2024). Novel porous hydrogel beads based on amidoxime modified polymer of intrinsic microporosity for efficient cationic dye removal. Microporous Mesoporous Mater..

[35] Zheng A.L.T., Phromsatit T., Boonyuen S., Andou Y. (2020). Synthesis of silver nanoparticles/porphyrin/reduced graphene oxide hydrogel as dye adsorbent for wastewater treatment. FlatChem.

[36] Hou N., Wang R., Wang F., Bai J.H., Jiao T.F., Bai Z.H., Zhang L.X., Zhou J.X., Peng Q.M. (2019). Self-assembled hydrogels constructed via host-guest polymers with highly efficient dye removal capability for wastewater treatment. Colloids Surf. A Physicochem. Eng. Asp..

[37] Moharrami P., Motamedi E. (2020). Application of cellulose nanocrystals prepared from agricultural wastes for synthesis of starch-based hydrogel nanocomposites: Efficient and selective nanoadsorbent for removal of cationic dyes from water. Bioresour. Technol..

[38] Peng N., Hu D.N., Zeng J., Li Y., Liang L., Chang C.Y. (2016). Superabsorbent Cellulose-Clay Nanocomposite Hydrogels for Highly Efficient Removal of Dye in Water. ACS Sustain. Chem. Eng..

[39] Han D.X., Zhao H.J., Gao L.L., Qin Z.H., Ma J.M., Han Y., Jiao T.F. (2021). Preparation of carboxymethyl chitosan/phytic acid composite hydrogels for rapid dye adsorption in wastewater treatment. Colloids Surf. A Physicochem. Eng. Asp..

[40] Far B.F., Naimi-Jamal M.R., Jahanbakhshi M., Khalafvandi S.A., Alian M., Jahromi D.R. (2024). Decontamination of Congo red dye from aqueous solution using nanoclay/chitosan-graft-gelatin nanocomposite hydrogel. J. Mol. Liq..

[41] de Araujo C.M.B., Ghislandi M.G., Rios A.G., da Costa G.R.B., do Nascimento B.F., Filipe A., Ferreira P., Sobrinho M.A.D., Rodrigues A.E. (2022). Wastewater treatment using recyclable agar-graphene oxide biocomposite hydrogel in batch and fixed-bed adsorption column: Bench experiments and modeling for the selective removal of organics. Colloids Surf. A Physicochem. Eng. Asp..

[42] Lv A., Lv X., Xu X., Chen Y., Zhang J., Shao Z.-B. (2024). Tailored multifunctional composite hydrogel based on chitosan and quaternary ammonium ionic liquids@montmorillonite with different chain lengths for effective removal of dyes and 4-nitrophenol. Sep. Purif. Technol..

[43] Rahul, Jindal R. (2024). Efficient removal of toxic dyes malachite green and fuchsin acid from aqueous solutions using Pullulan/CMC hydrogel. Polymer.

[44] Jaymand M. (2024). Biosorptive removal of cationic dyes from ternary system using a magnetic nanocomposite hydrogel based on modified tragacanth gum. Carbohydr. Polym. Technol. Appl..

[45] Altaleb H.A. (2024). Effective removal of hazardous cationic dye from polluted water using sulfonated copolymer hydrogel: Synthesis, nonlinear isotherm, and kinetics investigation. J. Saudi Chem. Soc..

[46] Jana S., Ray J., Mondal B., Pradhan S.S., Tripathy T. (2018). pH responsive adsorption/desorption studies of organic dyes from their aqueous solutions by katira gum-cl-poly(acrylic acid-co-N-vinyl imidazole) hydrogel. Colloids Surf. A Physicochem. Eng. Asp..

[47] Lamkhao S., Tandorn S., Rujijanagul G., Randorn C. (2023). A practical approach using a novel porous photocatalyst/hydrogel composite for wastewater treatment. Mater. Today Sustain..

[48] Das T., Patel D.K. (2024). Efficient removal of cationic dyes using lemon peel-chitosan hydrogel composite: RSM-CCD optimization and adsorption studies. Int. J. Biol. Macromol..

[49] Huaman M.A.L., Manco A.E.Q., López F., Carrasco R.L.A., Chacón A.M.L., Khan S. (2024). Removal of methylene blue dye from water with Fe_3_O_4_/poly(HEMA-co-AMPS) magnetic hydrogels. Results Chem..

[50] Azzeddine T., Marrane S.E., Goudali O., El Kaim Billah R., Boudouma A., Chaouiki A., Soufiane A., Agunaou M., Bahsis L. (2024). Simple and modified chitosan gel beads from a natural source as a bio-sorbent for water defluoridation: Experimental and computational perspectives. Inorg. Chem. Commun..

[51] Jaques L.L., Malheiro W.C., Jensen A.T., Machado F. (2024). Insights into the synthesis of hydrogels containing glycerol-based macromonomers for wastewater treatment: Focus on the efficient extraction of caffeine and mercury. J. Environ. Chem. Eng..

[52] Li M., Zhang P., Mao J., Li J., Zhang Y., Xu B., Zhou J., Cao Q., Xiao H. (2024). Construction of cellulose-based hybrid hydrogel beads containing carbon dots and their high performance in the adsorption and detection of mercury ions in water. J. Environ. Manag..

[53] Chu Q.K., Liu Z.X., Feng F., Chen D.L., Qin J., Bai Y.F., Feng Y. (2024). A novel bio-based fluorescent N, P-CDs@CMC/PEI composite hydrogel for sensitive detection and efficient capture of toxic heavy metal ions. J. Hazard. Mater..

[54] Thiyagarajan M., Pazhanisamy P., Gomathi T., Radha E., Vijayakumar S. (2024). Chromium adsorption studies of CaCO_3_ intercalated N-tert-amyl acrylamide-co-acrylamide/AMPS hydrogels. Inorg. Chem. Commun..

[55] Ouass A., Kadiri L., Hsissou R., El Amri A., Lebkiri I., Abbou B., Lebkiri A., Rifi E.H. (2024). Efficient removal of chromium (III) ions from aqueous solutions using sodium polyacrylate hydrogel powder: Characterization, kinetics, and regeneration studies. Inorg. Chem. Commun..

[56] Zhao H., Li Y. (2021). Removal of heavy metal ion by floatable hydrogel and reusability of its waste material in photocatalytic degradation of organic dyes. J. Environ. Chem. Eng..

[57] Li Y., Xie L.Y., Qu G., Zhang H., Dai Y.M., Tan J.L., Zhong J.R., Zhang Y.F. (2024). Efficient treatment of palladium from wastewater by acrolein cross-linked chitosan hydrogels: Adsorption, kinetics, and mechanisms. Int. J. Biol. Macromol..

[58] Chen Y.N., Li L.S.Z., Li Y.P., Liu Y.H., Chen Y.R., Li H., Li M.L., Xu F.T., Liu Y.Q. (2021). Preparation of a double-network hydrogel based on wastepaper and its application in the treatment of wastewater containing copper(ii) and methylene blue. RSC Adv..

[59] Kamal K.K., Hassan M.A., Kamel S., El-Sayed N.S. (2024). Efficient removal of Cd^2+^ ions and methylene blue from aqueous solutions by polyanionic sodium alginate-derived hydrogel. Surf. Interfaces.

[60] Jafarigol E., Ghotli R.A., Hajipour A., Pahlevani H., Salehi M.B. (2021). Tough dual-network GAMAAX hydrogel for the efficient removal of cadmium and nickle ions in wastewater treatment applications. J. Ind. Eng. Chem..

[61] Chen F., Zhao Y., Zhao H., Zhou X., Liu X. (2024). Heavy Metal Removal from Wastewater Using Poly(Gamma-Glutamic Acid)-Based Hydrogel. Gels.

[62] Mu R.H., Liu B., Chen X., Wang N., Yang J. (2020). Hydrogel adsorbent in industrial wastewater treatment and ecological environment protection. Environ. Technol. Innov..

[63] Zhang H.P., Tang P.F., Yang K., Wang Q.Y., Feng W., Tang Y.H. (2023). PAA/TiO_2_@C composite hydrogels with hierarchical pore structures as high efficiency adsorbents for heavy metal ions and organic dyes removal. Desalination.

[64] Sun J.H., Hu R.M., Zhao X.X., Liu T., Bai Z.S. (2024). A novel chitosan/cellulose phosphonate composite hydrogel for ultrafast and efficient removal of Pb(II) and Cu(II) from wastewater. Carbohydr. Polym..

[65] Niu H.Y., Li J.C., Li J.S., Yi C., Niu C.G. (2023). Preparation, properties and applications of porous hydrogels containing thiol groups for heavy metal removal. J. Environ. Chem. Eng..

[66] Ma M.Y., Ke X., Wang T., Li J., Ye H.P. (2024). A novel double-network hydrogel made from electrolytic manganese slag and polyacrylic acid-polyacrylamide for removal of heavy metals in wastewater. J. Hazard. Mater..

[67] Yang L.Z., Bao L., Zhong Y., Hao C., Chen J.J., Wu J.B., Wang X.H. (2024). Fabrication of in situ metal-organic framework grown on sodium lignosulphonate hydrogel for removal of Pb^2+^, methylene blue and crystal violet from aqueous solution. J. Clean. Prod..

[68] Zhang S.P., Ding J., Tian D.Y., Su W.H., Liu F.F., Li Q.L., Lu M.H. (2024). Preparation of novel poly(sodium p-styrenesulfonate)/sodium alginate hydrogel incorporated with MOF-5 nanoparticles for the adsorption of Pb(II) and tetracycline. J. Mol. Struct..

[69] Yang H.M., Wang S.C., Liu Y.X., Hu Y., Shen W.B. (2024). ZIF-67 grows in chitosan-rGO hydrogel beads for efficient adsorption of tetracycline and norfloxacin. Sep. Purif. Technol..

[70] Ghazy O., Hamed M.G., Breky M., Borai E.H. (2021). Synthesis of magnetic nanoparticles-containing nanocomposite hydrogel and its potential application for simulated radioactive wastewater treatment. Colloids Surf. A Physicochem. Eng. Asp..

[71] Dai Z.R., Wu H.A., Chen L.J., Gao Y., Li L., Ding D.X. (2024). Phytic acid-functionalized polyamidoxime/alginate hydrogel for targeted uranium extraction from acidic wastewater. Carbohydr. Polym..

[72] Yang J.J., Nie J.A., Bian L., Zhang J.M., Song M.X., Wang F., Lv G.C., Zeng L., Gu X.B., Xie X. (2024). Clay minerals/sodium alginate/polyethylene hydrogel adsorbents control the selective adsorption and reduction of uranium: Experimental optimization and Monte Carlo simulation study. J. Hazard. Mater..

[73] Salahuddin B., Aziz S., Gao S., Hossain M.S.A., Billah M., Zhu Z., Amiralian N. (2022). Magnetic Hydrogel Composite for Wastewater Treatment. Polymers.

[74] Jia J., Zhu J.L., Guo L.M., Yu J.Y., Li J., Li F.X. (2024). Synthesis and characterization of a β-cyclodextrin-MOF-based porous hydrogel for efficient adsorption of Au^3+^, Ag^+^, and Pb^2+^ ions. Sep. Purif. Technol..

[75] Guo Y., Niu Z., Huang J., Ding Y., Li X., Song Y., Wen G., Li X. (2024). Bimetallic peroxide nanocatalytic gel for water disinfection. J. Environ. Chem. Eng..

[76] Zhang Y., Li K., Jun Liao J. (2020). Facile synthesis of reduced-graphene-oxide/rare-earth-metal-oxide aerogels as a highly efficient adsorbent for Rhodamine-B. Appl. Surf. Sci..

[77] He Z., Qin M., Han C., Bai X., Wu Y., Yao D., Zheng Y. (2022). Pectin/graphene oxide aerogel with bamboo-like structure for enhanced dyes adsorption. Colloids Surf. A Physicochem. Eng. Asp..

[78] Yadav S., Asthana A., Singh A.K., Patel J., Sreevidya S., Carabineiro S.A.C. (2022). Facile preparation of methionine-functionalized graphene oxide/chitosan polymer nanocomposite aerogel for the efficient removal of dyes and metal ions from aqueous solutions. Environ. Nanotechnol. Monit. Manag..

[79] Wang Q., Zhang X., Wang F., Xie Y., Wang C., Zhao J., Yang Q., Chen Z. (2021). Egg yolk/ZIF-8/CLPAA composite aerogel: Preparation, characterization and adsorption properties for organic dyes. J. Solid State Chem..

[80] Tao E., Ma D., Yang S.Y., Hao X. (2020). Graphene oxide-montmorillonite/sodium alginate aerogel beads for selective adsorption of methylene blue in wastewater. J. Alloys Compd..

[81] Zhang T., Xiao S., Fan K., He H., Qin Z. (2022). Preparation and adsorption properties of green cellulose-based composite aerogel with selective adsorption of methylene blue. Polymer.

[82] Jing K., Liu X., Liu T., Wang Z., Hui Liu H. (2023). Facile and green construction of carboxymethyl cellulose-based aerogel to efficiently and selectively adsorb cationic dyes. J. Water Process Eng..

[83] Wang Z., Song L., Wang Y., Zhang X.F., Yao J. (2021). Construction of a hybrid graphene oxide/nanofibrillated cellulose aerogel used for the efficient removal of methylene blue and tetracycline. J. Phys. Chem. Solids.

[84] Tang Z.W., Lin X.X., Chen Y.L., Pan Y.W., Yang Y.Q., Mondal A.K., Yu M.Q., Wu H. (2023). Preparation of mussel-inspired polydopamine-functionalized TEMPO-oxidized cellulose nanofiber-based composite aerogel as reusable adsorbent for water treatment. Ind. Crops Prod..

[85] Wang X., Xu Q., Zhang L., Pei L., Xue H., Li Z. (2023). Adsorption of methylene blue and Congo red from aqueous solution on 3D MXene/carbon foam hybrid aerogels: A study by experimental and statistical physics modeling. J. Environ. Chem. Eng..

[86] Hu X.D., Zhang T.Y., Yang B., Hao M., Chen Z.J., Wei Y., Liu Y.B., Wang X.X., Yao J.B. (2024). Water-resistant nanocellulose/gelatin biomass aerogel for anionic/cationic dye adsorption. Sep. Purif. Technol..

[87] Gong X.-L., Lu H.-Q., Li K., Li W. (2022). Effective adsorption of crystal violet dye on sugarcane bagasse–bentonite/sodium alginate composite aerogel: Characterisation, experiments, and advanced modelling. Sep. Purif. Technol..

[88] Li K., Lei Y., Liao J., Yong Zhang Y. (2021). A facile synthesis of graphene oxide/locust bean gum hybrid aerogel for water purification. Carbohydr. Polym..

[89] Joshi P., Sharma O.P., Ganguly S.K., Srivastava M., Khatri O.P. (2022). Fruit waste-derived cellulose and graphene-based aerogels: Plausible adsorption pathways for fast and efficient removal of organic dyes. J. Colloid Interface Sci..

[90] Wang Z., He X., Miao M., Xin Feng X. (2023). Recyclable adsorbent aerogels by in-situ growth of ZIF-8 on aramid nanofibers/poly(vinyl alcohol) for multiple water pollutants. Sep. Purif. Technol..

[91] Zhao J., He J., Liu L., Shi S., Guo H., Xie L., Chai X., Xu K., Du G., Zhang L. (2023). Self-cross-linking of metal-organic framework (MOF-801) in nanocellulose aerogel for efficient adsorption of Cr (VI) in water. Sep. Purif. Technol..

[92] Li Y., Liu H., Nie R., Li Y., Li Q., Lei Y., Guo M., Zhang Y. (2024). Highly efficient adsorption of anionic dyes on a porous graphene oxide nanosheets/chitosan composite aerogel. Ind. Crops Prod..

[93] Ma L., Li D., Chen X., Xu H., Tian Y. (2024). A sustainable carbon aerogel from waste paper with exceptional performance for antibiotics removal from water. J. Hazard. Mater..

[94] Hadi Yatimzade M., Ahmadpour A., Ghahramaninezhad M., Moatamed Sabzevar A. (2024). Optimizing the efficient removal of ibuprofen from water environment by magnetic carbon aerogel: Kinetics, isotherms, and thermodynamic studies. J. Mol. Liq..

[95] Lentz L., Mayer D.A., Dogenski M., Ferreira S.R.S. (2022). Hybrid aerogels of sodium alginate/graphene oxide as efficient adsorbents for wastewater treatment. Mater. Chem. Phys..

[96] Yin J., Huang G., Xiao H., Chen N., An C., Chao T.C., Feng R., Read S. (2023). Bioinspired and dual-functional nanocellulose aerogels for water disinfection and heavy metal removal. Nano Today.

[97] Nguyen T.H.T., Nguyen K.T., Le B.H., Nghiem X.T., La D.D., Nguyen D.K., Nguyen H.P.T. (2024). Synthesis of magnetic Fe_3_O_4_/graphene aerogel for the removal of 2,4-dichlorophenoxyacetic acid herbicide from water. RSC Adv..

[98] Zhu H., Chen S., Luo Y. (2023). Adsorption mechanisms of hydrogels for heavy metal and organic dyes removal: A short review. J. Agric. Food Res..

[99] Hasanpour M., Hatami M. (2020). Application of three dimensional porous aerogels as adsorbent for removal of heavy metal ions from water/wastewater: A review study. Adv. Colloid Interface Sci..

[100] Ragitha V.M., Edison L.K., Thomas S., AR A., Jose Chirayil C., Thomas B. (2022). Safety Issues, Environmental Impacts, and Health Effects of Biopolymers. Handbook of Biopolymers.

